# The hospital costs of treating lung cancer in the United Kingdom

**DOI:** 10.1038/sj.bjc.6690341

**Published:** 1999-04

**Authors:** J L Wolstenholme, D K Whynes

**Affiliations:** Trent Institute for Health Services Research, University Medical School, Queen's Medical Centre, Nottingham NG7 2UH, UK; Department of Economics, University of Nottingham, Nottingham NG7 2RD, UK

**Keywords:** cancer, costs, lung cancer

## Abstract

A detailed patient-by-patient costing analysis, based on case records for 253 patients diagnosed in 1993, reveals that the mean 4-year diagnosis and management costs amounted to £6150 and £5668 for non-small cell and small cell lung cancer respectively. These costs are lower than those identified in Canadian studies, the difference being explained by the use of a simulated costing methodology in these studies, lower unit costs and less aggressive interventions. © 1999 Cancer Research Campaign


*British Journal of Cancer* (1999) **80**(1/2), 215–218
© 1999 Cancer Research Campaign
Article no. bjoc.1998.0341

### The hospital costs of treating lung cancer in the United Kingdom

**JL Wolstenholme[1] and DK Whynes[2]**

[1]Trent Institute for Health Services Research, University Medical School, Queen's Medical Centre, Nottingham NG7 2UH, UK; [2]Department of Economics, University of Nottingham, Nottingham NG7 2RD, UK

**Summary** A detailed patient-by-patient costing analysis, based on case records for 253 patients diagnosed in 1993, reveals that the mean 4-year diagnosis and management costs amounted to £6150 and £5668 for non-small cell and small cell lung cancer respectively. These costs are lower than those identified in Canadian studies, the difference being explained by the use of a simulated costing methodology in these studies, lower unit costs and less aggressive interventions.

**Keywords:** cancer; costs; lung cancer

As is the case for most industrialized countries, lung cancer is the most prevalent form of cancer in the UK. Along with ischaemic heart disease, cerebrovascular disease and pneumonia, it has ranked amongst the top four causes of mortality over the past 3 decades (Office of Health Economics, 1997). Although it is generally believed that lung cancer treatment places a substantial burden on national health care resources, few data are available to substantiate this belief (Evans et al, 1995*a*). This paper presents estimates of the direct economic costs of the hospital treatment of lung cancer, based on the records of a sample of patients drawn from the Trent region (central England).

Most cases of lung cancer present symptomatically, either in general practice or as emergency hospital admissions. Diagnosis is usually made on the basis of clinical examination and chest X-ray, complemented with, for example, bronchoscopy, needle biopsy, lung function tests, computerized tomography and/or forms of radioisotope scanning. The majority of lung cancer patients (typically around 75–80%) are diagnosed with non-small cell lung cancer (NSCLC) (Report of a meeting of physicians at the Royal Marsden Hospital, 1995). For them, surgical techniques may prove appropriate, whilst inoperable patients will be treated with radiotherapy (Williams, 1992) and, rarely in the UK at present, chemotherapy. Chemotherapy is the preferred treatment for small cell lung cancer (SCLC) cases, on occasions in combination with radiotherapy (Standing Medical Advisory Committee, 1994). Patients will usually receive post-treatment follow-up involving X-rays and/or scanning, although practices and policies appear to vary widely between clinics (Virgo et al, 1996). The majority of patients will eventually require palliative chemotherapy, palliative radiotherapy and/or symptom control. This having been said, it is probable that many patients never receive any treatment for lung cancer.

*Received 12 March 1998*
*Revised 16 October 1998*
*Accepted 4 November 1998*

*Correspondence to:* DK Whynes

### MATERIALS AND METHODS

The epidemiology of cancer in the UK has long been documented by the regional Cancer Registries, although the Registry databases do not include details of resource use. Our cost estimates were therefore based on an analysis of treatment records of a sample of individual patients. Individual patient costing provides far more realistic overall cost estimates than average costing or simulated costing. However, by auditing cost-events for each single patient, the procedure is particularly time-consuming (Whynes and Walker, 1995). With 3–4000 lung cancer cases being diagnosed annually in Trent, a complete cost analysis of a full year's cohort was deemed to be prohibitively expensive. Accordingly, we identified an approximately 1-in-10, random sample of 300 cancer patients from the Trent register. All had been nominally diagnosed with lung cancer in 1993. We selected the particular year as a baseline on the grounds that it would permit us to construct a resource audit for each patient for up to 4 years, a period following diagnosis during which the majority of cancer recurrences and treatment complications would be likely to occur. An attempt to retrieve the full treatment records of these cases revealed that, for 47 patients, notes were untraceable, or cancers had been misclassified, either by diagnosis or by year. After these exclusions, 253 records were available for analysis.

A full audit of resource-using hospital events was compiled for each of these patients, for 4 years following initial diagnosis or until death, if occurring earlier. The unit costs of these events were obtained from a survey of 11 of the region's principal service providers, each of whom was sent a form requesting the cost of the various activities, as performed at their site. Since 1993, all National Health Service (NHS) providers have been required to follow a uniform accounting protocol, requiring that their services be costed at full cost, i.e. all service-specific variable costs, with the inclusion of the relevant components of fixed and overhead costs (NHS Management Executive, 1993). We employed the mean of the reported costs of each event in our estimates, converted back to 1993 prices using the NHS pay and price index. Given that management events were occurring across time, the costs of events occurring in years 2 through 4 following diagnosis were discounted at 6%. In other words, mean 4-year costs were expressed as a 1993 present value, to represent the prospective cost implications from the perspective of the baseline year. The discount rate chosen was that conventionally employed in evaluations of UK public sector projects (Parsonage and Neuberger, 1992).

### RESULTS

Of the random sample selected for analysis, approximately 10% of cases (26/253) were diagnosed as SCLC. The mean/median age at diagnosis of the samples of patients was 71/72 years [standard deviation (s.d.) 9, range 35 years] for NSCLC, and 66/67 years (s.d. 9, range 47 years) for SCLC. Considerable diversity with respect to cost-events was evident on a patient-by-patient basis. For example, a total of 19 distinct diagnostic events were found to have occurred amongst the sample as a whole, and in a wide variety of combinations. Table 1 displays data on the frequency of use of investigations and treatments for the first year of diagnosis and treatment only, together with mean unit costs derived from the provider survey. Surgery included thoracotomy, lobectomy, segmentectomy and pneumonectomy. In most cases, many or all of the diagnostic events were undertaken on an in-patient basis although, in others, some of the tests were administered on an out-patient or day-case basis, with differential consequences for costs. Table 2 displays data pertaining to usage of in-patient care over the entire 4-year period. On the basis of our provider survey, the mean overhead cost of in-patient care amounted to £186.20 per day. Our estimate for in-patient palliative care comprises only hospital care for palliation related directly to lung cancer and associated events, for example, metastases.

Patient-specific radiotherapy costs varied with type (palliative or radical, low energy or simple), number of fractions and setting (e.g. in-patient or out-patient), with mean costs per episode being within a very wide range, £290–6650. Within the sample, seven different chemotherapy drugs were employed, in combinations, dosages and settings specific to each of the patients so treated. Again, unit drug costs were highly variable; for example, cisplatin at £0.5 per ml, methotrexate at £1 per ml and mitomycin at £2 per mg (all approximate). After primary treatment, each patient received one or more of up to 13 forms of immediate procedure or follow-up investigation, largely the same as those used as initial investigations (Table 1). Twenty per cent of NSCLC, and 23% of SCLC, patients required further in-patient stays, including emergency admission, surveillance for metastases, spinal cord compression, pleural effusion and blood transfusion.

In consequence of the great diversity in patient management routes, the cost audits of patient-specific events rapidly assumed great complexity. Space precludes a full exposition of the patient-specific cost algorithms developed, although these are available from the authors on request.

Table 3 displays the mean 4-year costs by broad management category. Costs associated with in-patient episodes accounted for 80% and 76% of mean costs for NSCLC and SCLC respectively. Only 14 patients (6%) survived the full 4 years following diagnosis and, on average, 96% and 97% of all costs were incurred in the first year in the two sub-samples respectively. In consequence, the cost estimates were extremely insensitive to variations in the discount rate. It is evident from Table 3 that, for both the cost totals and the majority of management categories, the means exceeds the medians, implying distributional skews to the right. Evidently, a small numbers of patients generated disproportionately high costs, a conclusion supported by the high value for

**Table 1** Utilization of services and unit costs during first year following diagnosis

| | Non-small cell (*n* = 227) | | Small cell (*n* = 26) | | |
|---|---|---|---|---|---|
| | Number | % | Number | % | Unit costs (£) |
| Diagnosis and initial investigations | | | | | |
| Chest X-ray | 215 | 94.7 | 26 | 100.0 | 11.5 |
| Blood count | 205 | 90.3 | 23 | 88.5 | 3.3 |
| Liver function test | 143 | 63.0 | 16 | 61.5 | 6.6 |
| Bronchoscopy | 128 | 56.4 | 18 | 69.2 | 308.4 |
| Lung function test | 112 | 49.3 | 7 | 26.9 | 79.8 |
| CT scan | 97 | 42.7 | 12 | 46.2 | 94.3 |
| Biopsy (lung) | 94 | 41.4 | – | – | 42.3 |
| Sputum cytology | 67 | 29.5 | 9 | 34.6 | 13.7 |
| Ultrasound | 43 | 18.9 | 9 | 34.6 | 27.9 |
| Other X-ray | 27 | 11.9 | 2 | 7.7 | 21.4 |
| Bone scan | 21 | 9.3 | 3 | 11.5 | 77.0 |
| Pleural aspiration | 19 | 8.4 | 3 | 11.5 | 45.0 |
| Percutaneous needle biopsy | 15 | 6.6 | 1 | 3.8 | 67.9 |
| Fine needle aspiration | 13 | 5.7 | – | – | 43.1 |
| Biopsy (lymph node) | 8 | 3.5 | 4 | 15.4 | 42.3 |
| Biopsy (pleural) | 8 | 3.5 | 1 | 3.8 | 42.3 |
| Mediastinoscopy | 4 | 1.8 | – | – | 239.5 per day |
| MRI scan | 3 | 1.3 | 1 | 3.8 | 163.6 |
| Bone marrow aspiration | 2 | 0.9 | 1 | 3.8 | 45.0 |
| Treatment | | | | | |
| Inpatient palliative care | 92 | 40.5 | 10 | 38.5 | 231.6 per day |
| Palliative radiotherapy | 79 | 34.8 | 13 | 50.0 | see text |
| Surgery | 17 | 7.5 | – | – | 382.1 per day |
| Radical radiotherapy | 9 | 4.0 | 2 | 7.7 | see text |
| Chemotherapy | 4 | 1.8 | 15 | 57.7 | see text |

© Cancer Research Campaign 1999

**Table 2** Length of in-patient stay (days)

| | Non-small cell | | | | Small cell | | | |
|---|---|---|---|---|---|---|---|---|
| | Patients receiving: | | for which: | | Patients receiving: | | for which: | |
| | Number | % | Mean | s.d. | Number | % | Mean | s.d. |
| Diagnosis | 168 | 74.0 | 13.1 | 15.0 | 20 | 76.9 | 11.2 | 8.6 |
| Surgery | 17 | 7.5 | 13.7 | 6.3 | 0 | – | – | – |
| Radical radiotherapy | 3 | 1.3 | 14.7 | 9.1 | 0 | – | – | – |
| Chemotherapy | 3 | 1.3 | 4.0 | 2.0 | 5 | 19.2 | 15.4 | 9.2 |
| Palliative radiotherapy | 18 | 7.9 | 13.2 | 16.8 | 0 | – | – | – |
| Inpatient palliative care | 104 | 45.8 | 25.1 | 48.3 | 11 | 42.3 | 13.7 | 11.3 |
| Further investigations | 44 | 19.4 | 12.5 | 12.9 | 6 | 23.1 | 6.3 | 5.6 |

**Table 3** Four-year costs of lung cancer diagnosis, treatment and follow-up (£ in 1993)

| | Patients receiving: | | for which: | Distribution at: | | | |
|---|---|---|---|---|---|---|---|
| | Number | % | Mean | 25% | 50% | 75% | Range |
| Non-small cell | | | | | | | |
| Diagnosis | 227 | 100.0 | 2954 | 555 | 1673 | 4667 | 28 695 |
| Surgery | 17 | 7.5 | 5230 | 3248 | 5350 | 6878 | 8025 |
| Radical radiotherapy | 10 | 4.4 | 4345 | 634 | 2444 | 11 029 | 10 947 |
| Chemotherapy | 4 | 1.8 | 945 | 319 | 719 | 1797 | 1733 |
| Palliative radiotherapy | 82 | 36.1 | 727 | 77 | 291 | 772 | 6581 |
| Inpatient palliative care | 93 | 41.0 | 3962 | 559 | 1862 | 3723 | 67 952 |
| Further investigations | 44 | 19.4 | 2958 | 1117 | 2048 | 4448 | 10 989 |
| Follow-up | 120 | 52.9 | 387 | 105 | 252 | 472 | 2824 |
| Total | 227 | 100.0 | 6150 | 1713 | 4132 | 8399 | 78 100 |
| Small cell | | | | | | | |
| Diagnosis | 26 | 100.0 | 2746 | 552 | 2287 | 4072 | 8585 |
| Surgery | 0 | 0.0 | – | – | – | – | – |
| Radical radiotherapy | 2 | 7.7 | 531 | 81 | 531 | – | 899 |
| Chemotherapy | 15 | 57.7 | 1558 | 100 | 381 | 2351 | 5707 |
| Palliative radiotherapy | 14 | 53.8 | 317 | 77 | 214 | 521 | 695 |
| Inpatient palliative care | 10 | 38.5 | 2649 | 1024 | 2445 | 3444 | 7633 |
| Further investigations | 6 | 23.1 | 2264 | 465 | 1582 | 3909 | 6513 |
| Follow-up | 17 | 65.4 | 414 | 114 | 280 | 671 | 1266 |
| Total | 26 | 100.0 | 5668 | 1810 | 4078 | 10 917 | 13 513 |

the range in some categories. Differences in both the mean and the median costs of the two lung cancer types were insignificant (Mann–Whitney test).

Patients' smoking histories should be routinely recorded in medical notes. Those recorded as being regular tobacco smokers at the time of admission constituted 43% of the sample. However, the mean 4-year management costs of this cohort did not differ significantly from those of non-smokers, those who had been recorded as never having smoked or having ceased smoking for at least 6 months prior to diagnosis.

### DISCUSSION

To date, the most ambitious attempt to cost lung cancer management has taken place in Canada (Rafuse, 1993; Evans et al, 1995*a*, 1995*b*, 1996). This research produced 5-year cost estimates of £10–15 000 for NSCLC and of £15–19 000 for SCLC (converted to sterling 1993, using the exchange rate and the NHS pay and price index). These estimates are considerably higher than those identified in our study, although the transparency of the Canadian research allows us to identify the sources of the discrepancies.

First, the assumed per diem cost of in-patient stay was almost twice as high in the Canadian study as it was in the Trent analysis. This presumably reflects on the different financial structures of the two health care systems and, as noted above, in-patient costs are a major component of overall management costs. Second, the methodology of the studies differs. The Canadian estimates have not been obtained solely from direct observation of patient experiences. They are based on simulations or models of events, derived both from agreed clinical protocols and from specialist opinion on the nature of 'proper practice'. In particular, the Canadian protocols appeared to have allowed for more radical treatments than was observed in our sample and the authors accepted that such an assumption might have been unrealistic in the case of elderly, frail patients. For example, over 85% of Canadian NSCLC patients were deemed eligible for surgical resection, with an average hospital stay of 20 days. In our sample, only 8% of NSCLC patients received resection, with an average stay of 14 days. Third, the Canadian study reported 5-year survival following diagnosis of all forms of lung cancer at 13%, superior to the observed 4-year rate for Trent. The proportion of costs incurred by the Canadian patients in the first year were 82% and 83% for NSCLC and SCLC respectively. These results are consistent with the view that the Trent patients received less aggressive therapy than was assumed to be the case for the Canadian patients.

The treatment costs of lung cancer may be compared directly with the equivalent costs estimated for two other sites. Mean 4-year costs for breast cancer treatment have been estimated at

© Cancer Research Campaign 1999 

£3–4000 for stages 1 to 3, but at approximately £6600 for stage 4 (Wolstenholme et al, 1998). Mean 5-year costs for cervical cancer management have been estimated at around £6600 for stage 1, but at £11–12 000 for stages 2 to 4 (Wolstenholme and Whynes, 1998). These three cost results are comparable as each of the studies concerned used the same estimation methods. As with the lung cancer case, the preponderance of resource use in both breast and cervical cancer occurred in the year immediately following diagnosis. These data suggest that the treatment costs of lung cancer are not disproportionately higher than for those for cancers at other sites.

### ACKNOWLEDGEMENTS

The research was supported by a grant from NHS Executive Trent. The authors gratefully acknowledge the assistance of Karine Thornhill and Sarah Smith with data collection.

### REFERENCES

Evans WK, Will BP, Bertholot J-M and Wolfson MC (1995*a*) The cost of managing lung cancer in Canada. *Oncology* **9**: 147–153

Evans WK, Will BP, Bertholot J-M and Wolfson MC (1995*b*) Diagnostic and therapeutic approaches to lung cancer in Canada and their costs. *Br J Cancer* **72**: 1270–1277

Evans WK, Will BP, Bertholot J-M and Wolfson MC (1996) The economics of lung cancer management in Canada. *Lung Cancer* **14**: 19–29

NHS Management Executive (1993) *Costing for Contracts Manual*. Department of Health: Leeds

Office of Health Economics (1997) *Compendium of Health Statistics*. OHE: London

Parsonage M and Neuberger H (1992) Discounting and health benefits. *Health Economics* **1**: 71–76

Rafuse J (1993) Study of lung cancer treatment costs may point to savings on a broader scale. *J Can Med Assoc* **149**: 1162–1165

Report of a meeting of physicians at the Royal Marsden Hospital U (1995) Small-cell lung cancer. *Lancet* **345**: 1285–1289

Standing Medical Advisory Committee (1994) *Management of Lung Cancer: Current Clinical Practices*. Department of Health: London

Virgo KS, Naunheim KS, McKirgan LW, Kissling ME, Lin JC and Johnson FE (1996) Cost of patient follow-up after potentially curative lung cancer treatment. *J Thoracic Cardiovasc Surg* **112**: 356–363

Whynes DK and Walker AR (1995) On approximations in treatment costing. *Health Economics* **4**: 31–39

Williams C (1992). *Lung Cancer – the Facts*. Oxford University Press: Oxford

Wolstenholme JL and Whynes DK (1998) Stage-specific treatment costs for cervical cancer in the United Kingdom. *Eur J Cancer* **34**: 1889–1893

Wolstenholme JL, Smith SJ and Whynes DK (1998) The costs of treating breast cancer in the United Kingdom: implications for screening. *Int J Technol Assessment Health Care* **14**: 277–289

*© Cancer Research Campaign 1999*
